# Draft genome sequences of four *Corynebacterium amycolatum* strains isolated from female urine samples

**DOI:** 10.1128/mra.00487-24

**Published:** 2024-07-31

**Authors:** Alex Kula, Grace Chilton, James Damaso, Yusef Golzar, Fatima Rushnaiwala, Helen Appleberry, Alan J. Wolfe, Catherine Putonti

**Affiliations:** 1 Department of Biology, Loyola University Chicago, Chicago, Illinois, USA; 2 Bioinformatics Program, Loyola University Chicago, Chicago, Illinois, USA; 3 Department of Microbiology and Immunology, Loyola University Chicago, Maywood, USA; University of Maryland School of Medicine, Baltimore, Maryland, USA

**Keywords:** *Corynebacterium amycolatum*, urinary tract infection, urobiome, OAB, rUTI

## Abstract

*Corynebacterium amycolatum* is an emerging pathogen of the urinary tract. Here, we present the draft genomes for four strains isolated from urine collected from symptomatic and asymptomatic female participants.

## ANNOUNCEMENT

Although *Corynebacterium amycolatum* is a commensal member of the skin flora, it is an opportunistic pathogen associated with cellulitis, endocarditis, peritonitis, and sepsis (1). Strains of this species also have been isolated from urinary samples (2) and posited to be an opportunistic pathogen of the urinary tract (3). To further investigate the genomic content of *C. amycolatum* strains from the urinary tract, here we present four genomes: UMB3517 and UMB3010, isolated from catheterized urine samples from females with overactive bladder (OAB) symptoms, UMB7578, isolated from a voided urine sample from a female diagnosed with recurrent urinary tract infections (rUTIs), and UMB0362, isolated from a catheterized urine sample from an asymptomatic (healthy) female.

Urine samples were collected as part of prior institutional review board (IRB) approved studies [see Table 1; (4–7)]. Strains were isolated from the sample using the expanded quantitative urine culture (EQUC) method (8) for the Loyola Urinary Education and Research Collaborative (LUEREC) collection. Prior to banking the strains, the genus and species were determined by matrix-assisted laser desorption ionization-time of flight (MALDI-TOF), as previously described (9). The frozen stock was obtained from the collection and streaked on a Brain Heart Infusion (BHI) agar plate and incubated at 35°C in 5% CO_2_ for 24 h. A single colony was selected and grown in liquid BHI medium under the same culture conditions. DNA was extracted using a DNeasy Blood and Tissue Kit (Qiagen), following the manufacturer’s instructions for Gram-positive organisms. Sequencing and library preparation were performed by SeqCoast Genomics (Portsmouth, NH). Samples were prepared with an Illumina DNA Prep tagmentation kit and sequenced with the Illumina NextSeq2000 platform (2 × 150 bp reads). Assemblies were performed using BV-BRC v3.35.5 (10) with the “auto” parameter. Briefly, the raw reads were trimmed using Trim Galore v0.6.5 (https://github.com/FelixKrueger/TrimGalore) and assembled using Unicycler v0.4.8 (11), followed by polishing with Pilon v1.23 (12). The genome assemblies were annotated using the BV-BRC Genome Annotation tool (10) and the NCBI Prokaryotic Genome Annotation Pipeline (PGAP) v6.7 (13); the latter is the publicly available annotation. Genome coverage, completeness, and contamination were calculated by BV-BRC. Virulence factor screening was performed using the VFanalyzer tool (14). Unless specified otherwise, default parameters were used for all software tools.

**TABLE 1:** Genome statistics and strain isolation source details

Strain	UMB0362	UMB3010	UMB3517	UMB7578
SRA Accession no.	SRR28710892	SRR28710896	SRR28710897	SRR28710893
Assembly Accession no.	JBCGES000000000	JBCGEV000000000	JBCGEW000000000	JBCGET000000000
No. of raw reads	2,044,004	1,780,282	2,784,774	2,504,768
Assembly length (bp)	2,503,085	2,456,972	2,501,019	2,554,220
G + C (%)	58.76	58.93	58.85	58.7
No. of contigs	53	44	32	49
Contigs N50 (bp)	127,036	107,160	216,091	135,435
Coverage (x)	115.19	102.27	146.26	135.60
Completeness (%)	100	100	100	100
Contamination (%)	0.4	0.6	0.4	0.6
Symptom status	Asymptomatic	OAB	OAB	rUTI
IRB no. (Institution)	LU206449 (LUC)	LU207152 (LUC)	LU207152 (LUC)	170077AW (UCSD)
Study reference	(7)	(4)	(4)	(5, 6)

Table 1 lists the genome assembly statistics for the four *C. amycolatum* strains. Virulence factor predictions for all four genome assemblies identified genes associated with siderophore-dependent iron uptake (*irp6A*, *irp6B*, and *irp6C*), regulation (*dtxR*, *mprB*, *senX3*, *sigA*/*proV*, and *whiB3*), and other virulence-associated classes. The virulence factor profiles for these four strains are congruent with those noted in the recent pangenomic study of the 26 publicly available genome assemblies for the species (15). The authors of this prior study suggest that these virulence factors were acquired via horizontal gene transfer, enabling the invasive phenotype observed in strains responsible for infections (15). Further sequencing and subsequent investigation of *C. amycolatum* genomes is needed to understand the prevalence of horizontal gene transfer in this species.

## Data Availability

Table 1 lists the SRA accession numbers and assembly accession numbers for the four strains.
